# Prenatal diagnosis of 7 cases with uniparental disomy by utilization of single nucleotide polymorphism array

**DOI:** 10.1186/s13039-021-00537-2

**Published:** 2021-03-19

**Authors:** Lili Zhou, Zhaoke Zheng, Yunzhi Xu, Xiaoxiao Lv, Chenyang Xu, Xueqin Xu

**Affiliations:** Center of Prenatal Diagnosis, Wenzhou Central Hospital, Wenzhou, 325000 People’s Republic of China

**Keywords:** uniparental disomy, single nucleotide polymorphism array, prenatal diagnosis

## Abstract

**Background:**

The phenotypes of uniparental disomy (UPD) are variable, which may either have no clinical impact, lead to clinical signs and symptoms. Molecular analysis is essential for making a correct diagnosis. This study involved a retrospective analysis of 4512 prenatal diagnosis samples and explored the molecular characteristics and prenatal phenotypes of UPD using a single nucleotide polymorphism (SNP) array.

**Results:**

Out of the 4512 samples, a total of seven cases of UPD were detected with an overall frequency of 0.16%. Among the seven cases of UPD, two cases are associated with chromosomal aberrations (2/7), four cases (4/7) had abnormal ultrasonographic findings. One case presented with iso-UPD (14), and two case presented with mixed hetero/iso-UPD (15), which were confirmed by Methylation-specific multiplex ligation-dependent probe amplification (MS-MLPA) as maternal UPD (15) associated with Prader-Willi syndrome (PWS). Four cases had iso-UPD for chromosome 1, 3, 14, and 16, respectively; this is consistent with the monosomy rescue mechanism. Another three cases presented with mixed hetero/isodisomy were consistent with a trisomy rescue mechanism.

**Conclusion:**

The prenatal phenotypes of UPD are variable and molecular analysis is essential for making a correct diagnosis and genetic counselling of UPD. The SNP array is a useful genetic test in prenatal diagnosis cases with UPD.

## Background

Uniparental disomy (UPD) is the presence of a homologous chromosomes, or segments of chromosomes, originated from the same parent [1]. UPD can be recognized as two subtypes of heterodisomy (hUPD)—inheritance of two homologous but genetically different chromosomes from one parent, and isodisomy (iUPD), which is the inheritance of two copies of one parental chromosome. UPD may comprise the whole chromosome, or just part of it (segmental UPD) There are three primary mechanisms by which UPD can occur: (i) trisomy rescue, where there is mitotic loss of the extra chromosome in the trisomy; (ii) monosomy rescue, where there is duplication of the single chromosome in the monosomy via non-disjunction; and (iii) gamete complementation, where a gamete is disomic for the same nullisomic chromosome of the second gamete, by chance [2].

UPD results in imprinting disorders and monogenetic disease-related disorders [3]. Thus far, only five chromosomes have been defined as imprinted based on the associated clinical phenotypes: chromosomes 6, 7, 11, 14, 15 and 20. Approximately 35% of karyotyped UPD cases are associated with chromosomal aberrations (e.g. mosaic triploidy, mosaic trisomy, small supernumerary marker chromosomes (sSMCs) and unbalanced translocation and duplication [4–8]. And 65% of UPD cases present with a normal karyotype, which cannot be identified by traditional karyotype analysis but can be confirmed by molecular markers or methylation patterns for the chromosomal region of interest.

Here, we characterized 7 cases of UPD by performing single-nucleotide polymorphism-based array. This study aimed to provide useful information for prenatal diagnosis of UPD and detailed genetic counselling.

## Results

### Frequency and chromosomal origin of UPD

We studied seven cases of loss of heterozygosity (LOH) greater than 20 Mb, both segmental and whole chromosome cases with an overall frequency of 0.16% (Table 1). There was no known history of parental consanguinity in these cases, and we hypothesize that the homozygosity is determined by UPD. UPD occurs on chromosomes 1, 3, 14, 15, and 16, respectively.

**Table 1:** Summary of karyotypes, SNP array result, ultrasound findings and pregnancy outcome

Case No	Specimen	Mechanism	Chromosome	Type	Segment	Size (Mb)	Mosaic% by SNP array	Karyotype	Origin	Indication
1	UCB	Monosomy rescue	Chr1	Mosaic UPD	p36.33q44	248.71	85	46,XX	Mitosis	Ventriculomegaly
2	AF	Monosomy rescue	Chr3	UPD	p26.3q29	197.59	100	46,XX	Mitosis	Hyperechogenic bowel
3	UCB	Monosomy rescue	Chr14	UPD	q11.2q32.33	86.49	100	46,XX	Mitosis	UMM
4	Villus	Monosomy rescue	Chr16	UPD	p13.3q24.3	90.16	100	47,XX, +20	Mitosis	IFD
		nondisjunction	Chr20	Trisomy	p13q13.33	62.89	100		Meiosis	
5	UCB	Trisomy rescue	Chr15	UPD	q22.2q26.3	39.48	100	46,XX	MI	UMM
6	UCB	Trisomy rescue	Chr15	UPD	q11.2q14	11.17	100	46,XY	MII	AMA
					q21.1q23	18.64	100			
7	AF	Trisomy rescue	Chr1	UPD	p36.33p13.2	115.03	100	47,XX, + mar[53]/46,XX[22]	MII	High T 21 risk
				UPD	q21.1q44	104.37	100			
				DUP	p13.2p11.2	5.39	100			

Results of seven cases of UPD

*UCB* Umbilical Cord blood, *AF* amniotic fluid, *MI* meiosis I, *MII* meiosis II, *UMM* Ultrasound multiple malformations, *IFD* intrauterine fetal death, *AMA* advanced maternal age, *T* trisomy

### Chromosomal aberrations and phenotypes of UPD

**Fig. 1:**
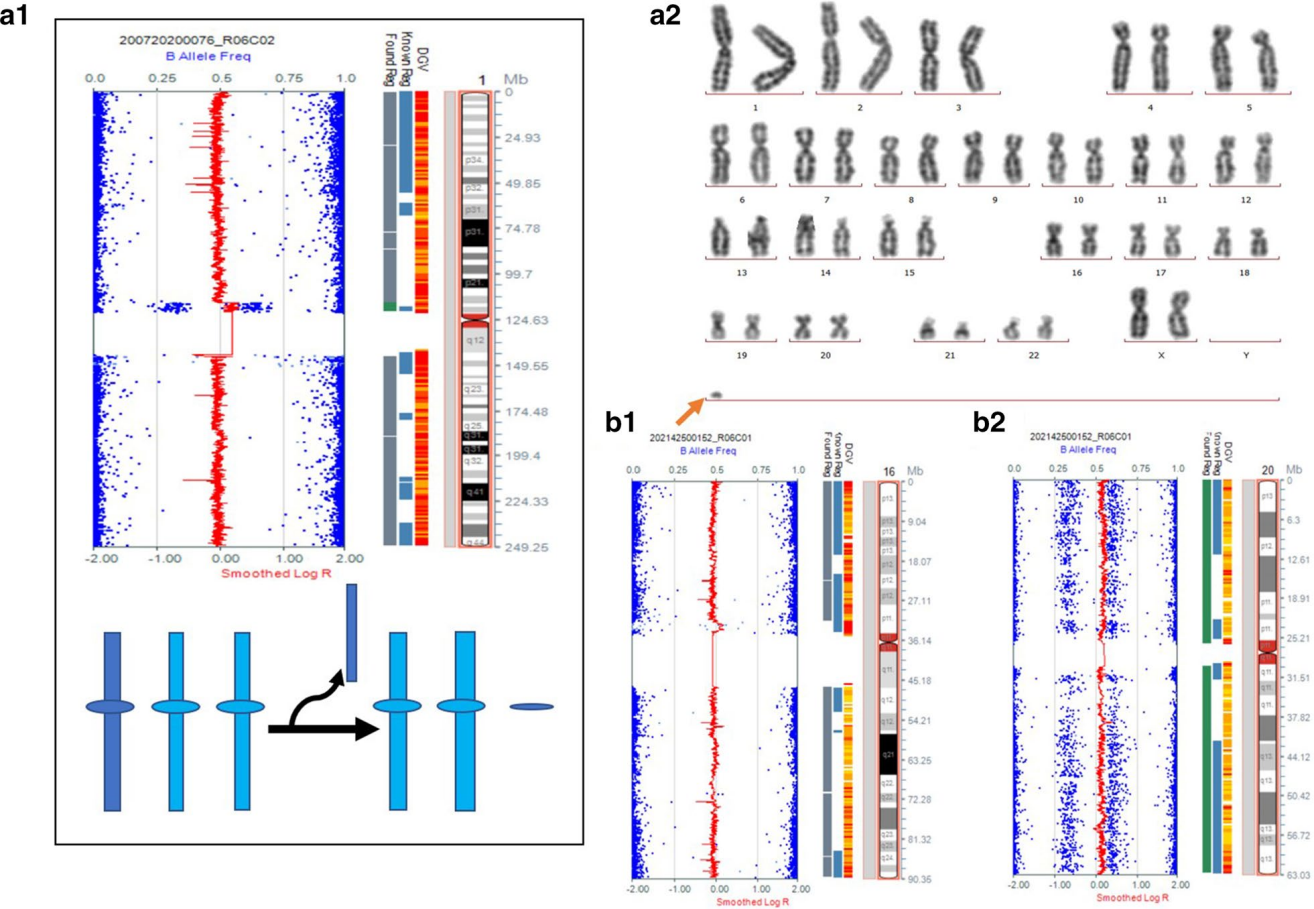
Cytogenetic and SNP array results of UPD combined with chromosomal aberrations. (**a1**) SNP array of case 7 was a partial duplication of chromosome 1 combined with UPD(1): arr 1p36.33p13.2(753541_115779865) × 2 hmz,1p13.2p11.2(115796490_121184898) × 3,1q21.1q44(144828599_249202755) × 2 hmz. (**a2**) G-banding of case 7 revealed the karyotype 47,XX, + mar[53]/46,XX[22]. (**b1, b2**) SNP array of case 4: arr 16p13.3q24.3(98642_90256266) × 2 hmz,(20) × 3

Among the seven cases of UPD, two cases are associated with chromosomal aberrations (2/7). One case had UPD combined with a small supernumerary marker chromosome, and one case had UPD combined with trisomy 20 (Fig. 1). The sSMC was trisomy for 5.39 Mb in the region 1p13.2p11.2 (chr1:115,796,490_121,184,898). We deemed the sSMC to be harmless, and UPD (1) to be a variant of unknown significance. The formation was likely via trisomy rescue, thus the women chose to continue the pregnancy. A girl was born naturally at 39 + 5 weeks and was followed-up for 2 years with development and growth both normal. Aside from the two cases, the other five cases of UPD were detected by SNP array but not by karyotype analysis.

The case of UPD combined with trisomy 20 resulted in intrauterine fetal death in the first trimester; the remaining six cases survived until the second or third trimester, indicating that UPD could be tolerated by the embryos. Four cases (4/6) had abnormal ultrasonographic findings.

### UPD related syndromes caused by imprinting

**Fig. 2:**
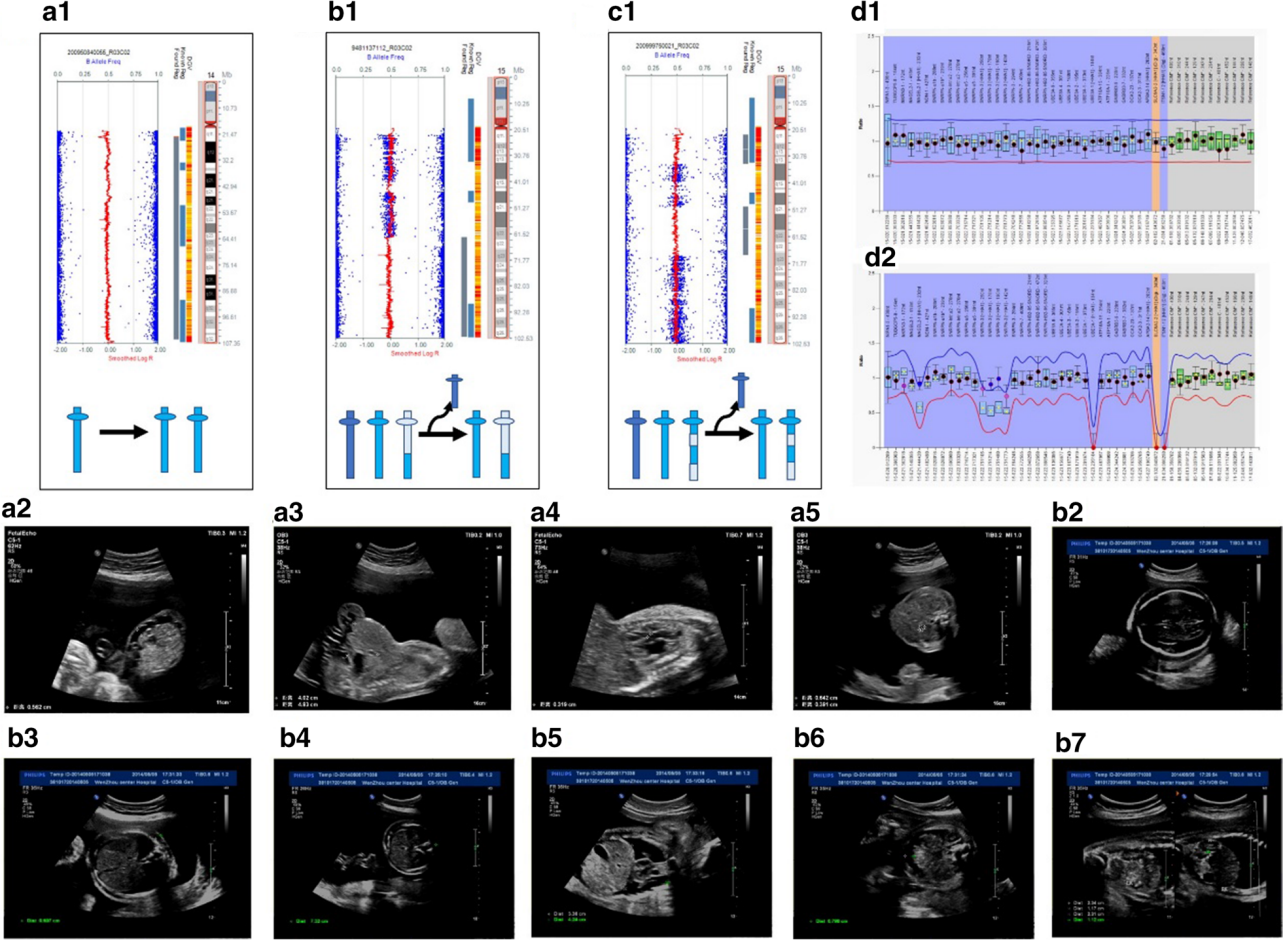
SNP array results and multiple ultrasonic malformations of three cases involved imprinted chromosome 14 and 15. (**a1**) SNP array revealed that case 3 arose by monosomy rescue with iso-UPD (14). (**a2**–**a5**) The multiple ultrasound malformations observed for case 3 included peritoneal effusion, omphalocele, ventricular septal defect, and small gastric vesicle. (**b1**) SNP array revealed that case 5 arose by trisomy rescue with mixed hetero/iso- UPD (15) from meiosis I non-disjunction error. (**b2**–**b7**) The multiple ultrasound malformations observed for case 5 included scalp edema, chest wall edema, abdominal wall edema, bilateral pleural effusion, ascites, and bilateral kidney edema. (**c1**) SNP array revealed that case 6 arose by trisomy rescue with mixed hetero/iso-UPD (15) from meiosis II nondisjunction error. (**d1**) The copy number ratio of the 15q11 region was 1 for two cases of UPD (15). (**d2**) Methylation ratio of the imprinted allele of chromosome 15q11 after digestion was 1 for two cases of UPD(15)

In total, three cases involved imprinted chromosome 14 and 15 (Fig. 2). iso-UPD(14) was identified in case 3 and was presumed to occur mitotically followed by monosomy rescue with normal karyotype results. Case 3 presented with multiple malformations on ultrasound, including peritoneal effusion, omphalocele, ventricular septal defect, and small gastric vesicle; this pregnancy was terminated at 34 + 3 weeks. Parental samples were not available for further tests to identify the UPD (14) parental origin. The couple decided to terminate the pregnancy at 34 + 3 weeks gestation.

Case 5 presented with mixed hetero/iso-UPD(15) of 15q22.2q26.3 with multiple malformations on ultrasound, including scalp edema, chest wall edema, abdominal wall edema, bilateral pleural effusion, ascites, and bilateral kidney edema; this pregnancy was terminated at 32 + 3 weeks. Case 6 presented with mixed hetero/iso-UPD(15) of 15q11.2q14 and 15q21.1q23 together with atrial septal defect; this pregnancy was not terminated. A boy was born naturally at 39 + 6 weeks and was followed-up for 12 months. He had hypotonia and difficulty feeding until nine months of age; after nine months, his feeding and appetite improved. He also had cryptorchidism. Those two cases of mixed hetero/iso-UPD (15) occurred meiotically followed by trisomy rescue, and were confirmed as maternal UPD (15) associated with PWS by MS-MLPA.

### Mechanism of UPD

Four cases had UPD for an entire chromosome, chromosome 1, 3, 14 and 16, respectively; this is consistent with the monosomy rescue mechanism. Another three cases were consistent with a trisomy rescue mechanism. One case of mixed hetero/iso-UPD (15) had heterozygous alleles near the centromere of chromosome 15, suggesting a meiosis I origin with one crossover of recombination. The other case of mixed hetero/iso-UPD (15) had LOH near the centromere and across the middle of the chromosome, suggesting meiosis II origin with three crossovers of recombination. Case 7 had homozygote alleles for chromosome 1 in its entirety combined with a 5.39 Mb size small supernumerary marker chromosome arising from meiotic II non-disjunction.

## Discussion

Here, we presented seven cases of UPD in 4512 prenatal cases using SNP array, with an overall frequency of 0.16%. UPD occurs on chromosomes 1, 3, 14, 15 and 16, respectively. The frequency of UPD cases has not yet been exactly definited in the general human population. Of 2019 patients with intellectual disabilities, developmental delay, abnormal growth, autism, and/or congenital abnormalities, UPD was detected in 0.54% [9]. Robinson detected the frequency of UPD in newborns is approximately 1 in 3500 [10]. UPD is also seen in different chromosome-specific frequencies. UPD (15) is present in 1 out of 80,000–100,000 births, paternal segmental UPD (11) is present in 1 out of 75,000 live births, and paternal UPD (6) is present in 1 out of 1,250,000 births.

Approximately 35% of karyotyped UPD cases are associated with chromosomal aberrations [4]. Some researchers have already found the frequencies of mosaic trisomy (39%) and small supernumerary marker chromosomes (17%) due to trisomic rescue, robertsonian (28%) and other translocations (6%), isochromosomes (3%), and other rearrangements correlated with UPD presence. Hence, UPD testing is advised when patients have chromosomal rearrangements (numerical and structural malformations) involving imprinting-related chromosomes. In our survey, two cases are associated with chromosomal aberrations (2/7, 28%).

The clinical phenotypes of UPD are variable, which range from unapparent to typical autosomal-recessive disease or syndromic imprinting disorder, depending on the parental origin and the specific chromosome or segment involved [11, 12]. In total, three cases of UPD involving imprinted chromosomes (14 and 15, respectively) were detected in this study. Chromosome 14 carries a 1 Mb cluster of imprinted genes located in 14q32, including paternally-expressed genes such as DLK1, RTL1, and DIO3, as well as maternally-expressed noncoding RNAs such as MEG3, RTL1as, MEG8, and numerous C/D box small nucleolar RNAs and microRNAs [13]. Maternal UPD (14) is associated with Temple syndrome, which is characterized by pre- and postnatal growth retardation, developmental delay, muscular hypotonia, joint laxity, small hands and feet, truncal obesity, precocious or early onset of puberty, and adult short stature [14]. Paternal UPD (14) is associated with Kagami–Ogata syndrome, which causes more serious phenotypes with polyhydramnios, thoracic dysplasia (coat hanger sign) with respiratory failure, abdominal defects, growth retardation, developmental delay, and facial abnormalities with full cheeks and protruding philtrum [15].

UPD (15) is associated with Prader Willi Syndrome (PWS) and Angelman syndrome (AS) which represent the best examples of genomic imprinting in humans. Two cases of UPD (15) in our cohort were confirmed as maternal UPD (15) associated with PWS by MS-MLPA. PWS is a multisystem disorder characterized by severe infantile hypotonia with poor suck and failure to thrive as well as hypogonadism. The estimated prevalence of PWS is 1/10,000–1/15,000 [16]. Central to the PWS region is the SNURF-SNRPN gene, which is unmethylated on the paternally-inherited expressed allele and methylated on the maternally-inherited repressed allele [17]. PWS occurs as a result of an absent expression of paternally-expressed imprinted genes at chromosome 15q11.2-q13 through paternal deletion of this region (65–75%), maternal UPD 15 (20–30%), or an imprinting defect (1–3%).

Different molecular approaches can be applied for UPD diagnostics, including microsatellite analyses, DNA-based methylation test, bisulfite sequencing, multiplex ligation-dependent probe amplification (MLPA), and SNP-array [18–21]. SNP microarrays have the advantage of detecting long continuous regions of homozygosity (ROH) in addition to chromosome CNV. One limitation of SNP microarray for diagnosing UPD is only able to detect iso-UPD but not hetero-UPD. Stephanie L. Santoro reported SNP microarray is likely able to detect over half of UPD (15), distinguish the specific subtype in approximately 80% of PWS [22]. Thus appropriate diagnostic algorithm is extremely important for laboratory testing of the UPD [23]. For example, for UPD (15), it is suggested that a DNA-based methylation test is first performed, which can detect more than 99% of individuals affected by PWS or Angelman syndrome (AS). DNA methylation analysis is the only technique that will diagnose PWS in all three molecular classes and differentiate PWS from AS in deletion cases, which is sufficient for clinical diagnosis and genetical counselling [24]. Interphase fluorescence in situ hybridization (FISH) analysis using corresponding specific probes should be followed, which can be replaced by SNP array as reported; if the latter does not detect a microdeletion in 15q11.2-12, a UPD test should be performed [25]. MLPA may be a good alternative for a quick and inexpensive test of an imprinting-related disorder [26]. The MS-MLPA assay combines both DNA methylation analysis and dosing analysis across the PWS region, and has been shown to investigate five distinct differentially methylated sites; it gives information on dosing in the 15q11.2 region [27]. However, UPD diagnosis by SNP array can be accomplished only if parental DNA is analyzed in the case of heterodisomy [9]. The limitation of our study that parental samples were not available for further tests to identify the parental origin of UPD remains.

## Conclusion

The prenatal phenotypes of UPD are variable and molecular analysis is essential for making a correct diagnosis and genetic counselling of UPD. The SNP array is a useful genetic test in prenatal diagnosis cases with UPD.

## Methods

### Study subjects

This study was approved by the institutional research ethics committee of Wenzhou Central Hospital. All patients agreed to participate in the study and provided written informed consent. We retrospectively analysed a cohort of 4512 prenatal samples referred for genome wide SNP array that were taken at the Wenzhou Prenatal Diagnosis Center between 2012 and 2018. The pregnant women ranged in age from 19 to 48 years, with their gestational week between 8 and 30 weeks. The indications for prenatal diagnostic testing included advanced maternal age, high-risk serological screening, abnormal non-invasive prenatal DNA test, ultrasonographic abnormal indications, either parent carrying chromosome abnormality, and history of intrauterine fetal death or aborted fetuses.

### SNP array analysis

Chromosomal microarray analysis was performed using the Illumina Human CytoSNP-12 array (Illumina, USA) according to the manufacturer’s instructions. The results were analyzed with Illumina BeadStudio software. All detected CNVs were compared with known CNVs in the scientific literature and publicly available databases: Database of Genomic Variants, DECIPHER database, International Standards for Cytogenomic Array, Online Mendelian Inheritance in Man and ClinGen Dosage Sensitivity Map. All reported copy number variants (CNVs) were based on the National Center for Biotechnology Information human genome build 37 (hg 19).

UPD changes were detected by assessing for aberrations in probe intensities (log R ratios) along with shifts in genotype frequencies of the SNP probes (B allele frequencies) [8]. UPD is diagnosed when the log R ratio is zero, which equates to two copies. Meanwhile, in UPD, the B allele frequency is 0% and 100%, and only two haplotypes can be seen. When UPD is visible near the telomeres, but not the centromere, meiosis I non-disjunction is indicated. When UPD is present at the centromeres, meiosis II non-disjunction is indicated. When UPD is present at the whole chromosome, mitosis non-disjunction is indicated.

### Methylation-specific multiplex ligation-dependent probe amplification (MS-MLPA) analysis

MS-MLPA probe sets and an ME028 Prader Willi/Angelman were supplied by MRC-Holland (http://www.mlpa.com). MS-MLPA analysis was performed according to the manufacturer’s instructions. Data were analyzed using the Coffalyser.NET software developed by the manufacturer. The Coffalyser.NET algorithm primarily runs two steps. First, the fluorescence of each probe is normalized against the reference probes within each reaction (both undigested and digested reactions). For calculation of copy number, relative probe signals from each undigested reaction of a test sample are compared with those obtained from the undigested reactions of reference samples. This comparison then allows for the determination of the relative copy number of the target sequences in a sample. For calculation of methylation status, the ratio obtained for each probe in the digested reaction is then compared to the ratio obtained in the corresponding undigested reaction. This ratio can be multiplied by 100 to give a methylation percentage. Finally, the methylation percentages in a test sample are compared to the percentages in the reference samples.

### Karyotype analysis

Culture: (i) Villi were digested to produce cell suspensions; the suspensions were centrifuged at 1200 r/min for 10 min. The supernatant was discarded after centrifugation, leaving about 1–2 mL of cell suspension. Then, 5 mL of amniocyte culture medium was added and the suspension was placed in an incubator at 37 °C with 5% CO_2_ for 9–10 days for growth. (ii) Twenty milliliters of amniotic fluid was centrifuged at 1200 r/min for 10 min. The supernatant was discarded after centrifugation, leaving about 1–2 mL of cell suspension. Then, 5 mL of amniocyte culture medium was added and the suspension was placed in an incubator at 37 °C with 5% CO_2_ for 9–10 days for growth. (iii) One milliliter of cord blood was added into lymphocyte culture medium and placed in an incubator at 37 °C with 5% CO_2_ for 68–72 h for growth.

Karyotype: Conventional G-banded karyotyping at 320–450 bands resolution was performed. Scanning was performed with a Leica GLS120 automated nuclear scanning system. Fifteen chromosome karyotypes were counted, and five karyotypes were analyzed by two doctors, according to the International System for Human Cytogenetic Nomenclature 2016 standard.

## Data Availability

All data generated or analyzed during this study are included in the article.

## Abbreviations

UPD: Uniparental disomy
SNP: Single nucleotide polymorphism
MS-MLPA: Methylation-specific multiplex ligation-dependent probe amplification
PWS: Pallister Killian syndrome
hUPD: Heterodisomy
iUPD: Isodisomy
sSMCs: Small supernumerary marker chromosomes
LOH: Loss of heterozygosity
AS: Angelman Syndrome
MLPA: Multiplex ligation-dependent probe amplification
FISH: Interphase fluorescence in situ hybridization
